# Increasing prevalence and burden of bronchiectasis in urban Chinese adults, 2013–2017: a nationwide population-based cohort study

**DOI:** 10.1186/s12931-022-02023-8

**Published:** 2022-05-04

**Authors:** Jingnan Feng, Lina Sun, Xiaoyan Sun, Lu Xu, Lili Liu, Guozhen Liu, Jinxi Wang, Pei Gao, Siyan Zhan, Yahong Chen, Shengfeng Wang, Yongchang Sun

**Affiliations:** 1 Department of Epidemiology and Biostatistics, School of Public Health, Peking University Health Science Center, Beijing, China; 2 Department of Pulmonary and Critical Care Medicine, Peking University Third Hospital, Beijing, China; 3 Peking University Health Information Technology Co. Ltd, Beijing, China; 4 Shanghai Songsheng Business Consulting Co. Ltd, Beijing, China; 5 Research Center of Clinical Epidemiology, Peking University Third Hospital, Beijing, China

**Keywords:** Bronchiectasis, Prevalence, Disease burden

## Abstract

**Background:**

While the prevalence and disease burden of bronchiectasis are increasing, data in the world’s largest population are lacking. We aimed to investigate the prevalence and disease burden of bronchiectasis in Chinese adults.

**Methods:**

We conducted a population-based study using data between 2013 and 2017 from the national databases of Urban Employee Basic Medical Insurance and Urban Resident Basic Medical Insurance in China. Data from over 380 million patients aged 18 years and older during the study period were analyzed, and a total of 383,926 bronchiectasis patients were identified. Primary outcomes included the age- and sex-specific prevalence of bronchiectasis. Annual visits and hospitalizations, as well as annual costs were also calculated.

**Results:**

The prevalence of bronchiectasis in Chinese adults increased 2.31-fold, from 75.48 (62.26, 88.69) per 100,000 in 2013 to 174.45 (137.02, 211.88) per 100,000 in 2017. The increase was more remarkable for patients aged over 50 years in both genders. The per-capita total cost and hospitalization cost of patients with bronchiectasis increased 2.18-fold and 1.83-fold from 2013 to 2017, respectively, mostly driven by non-bronchiectasis costs. The average annual hospitalization ranged from 1.20 to 1.24 times during the 5 years.

**Conclusion:**

The prevalence and disease burden of bronchiectasis in Chinese urban adults ≥ 18 years had increased significantly between 2013 and 2017.

**Supplementary Information:**

The online version contains supplementary material available at 10.1186/s12931-022-02023-8.

## Introduction

Bronchiectasis is a chronic respiratory disease characterized by a clinical syndrome of chronic productive cough and recurrent respiratory infections in the presence of abnormal and permanent dilation of the bronchi [1]. Recent studies provided evidence for increasing prevalence of bronchiectasis in adults in the UK and Germany [2, 3]. However, data also show large variations in estimates of the prevalence of bronchiectasis across studies [4–7], and some of which were based on smaller populations [7–10]. A cross-sectional questionnaire survey in China revealed a prevalence of patient-reported bronchiectasis of 1.2% in residents aged 40 years or older from 7 provinces/municipalities between 2002 and 2004 [7]. However, nationwide epidemiological data, particularly trends of the prevalence and disease burden of bronchiectasis, were lacking in the world’s largest population.

Bronchiectasis causes a significant burden on the healthcare system by frequent medical visits and hospitalizations. With increase in the prevalence of bronchiectasis in Germany [3], the overall age-adjusted rate of all bronchiectasis associated hospitalizations increased significantly between 2005 and 2011, and the length of hospital stay was comparable to that of chronic obstructive pulmonary disease (COPD), and longer than that of all hospitalizations [8]. Furthermore, the total direct expenditure per insured bronchiectasis patient during 2012 and 2015 was nearly one third higher than that for an age, gender and Charlson Comorbidity Index matched control [11]. However, data on hospitalization and medical costs of bronchiectasis from population-based studies were still rare, particularly in low- and middle-income countries. Therefore, we investigated the prevalence of bronchiectasis in urban adults and evaluated the hospitalization and cost burden of this disease in China from 2013 to 2017.

## Methods

### Data sources

Data on bronchiectasis were obtained from the Urban Employee Basic Medical Insurance (UEBMI) and Urban Resident Basic Medical Insurance (URBMI) schemes [12–14]. UEBMI covers working and retired employees in cities (i.e., employers and employees from government agencies and institutions, private businesses, social organizations, state-owned enterprises, and other private entities), and URBMI is for citizens without employment in cities (i.e., children, students, elderly people and unemployed residents). By 2016, UEBMI and URBMI had covered over 95% of the whole urban population. UEBMI and URBMI are updated on a monthly basis in all cities. Provinces/municipalities without detailed information on diagnosis were excluded. Data from about 0.5 billion residents in 23 provinces were included in this study. The variables included age, gender, International Classification of Diseases (ICD) code and name of primary and secondary diagnosis, type of medical visits, and total medical expenses. The study protocol was approved by the ethical review committee of Peking University Health Science Center, and the requirement for informed consent was waived (IRB00001052-18012).

### Study population

Data were analyzed over the 5-year period from January 1st, 2013 to December 31st, 2017. The time period of the study was chosen due to the availability of data. The inclusion criteria of the population were residents who (1) had either UEMBI or URBMI insurance during 2013 to 2017; (2) were enrolled in the 23 provinces included; and (3) aged ≥ 18 years in each year during the study period. Subjects with any one of the following were excluded: (1) without a valid national insurance ID; and (2) with conflicting information recorded, i.e., records of the medical treatment reimbursement time being earlier than the individual's first enrollment time. All claim records were anonymized to protect patients’ privacy.

### Bronchiectasis identification

**Fig. 1:**
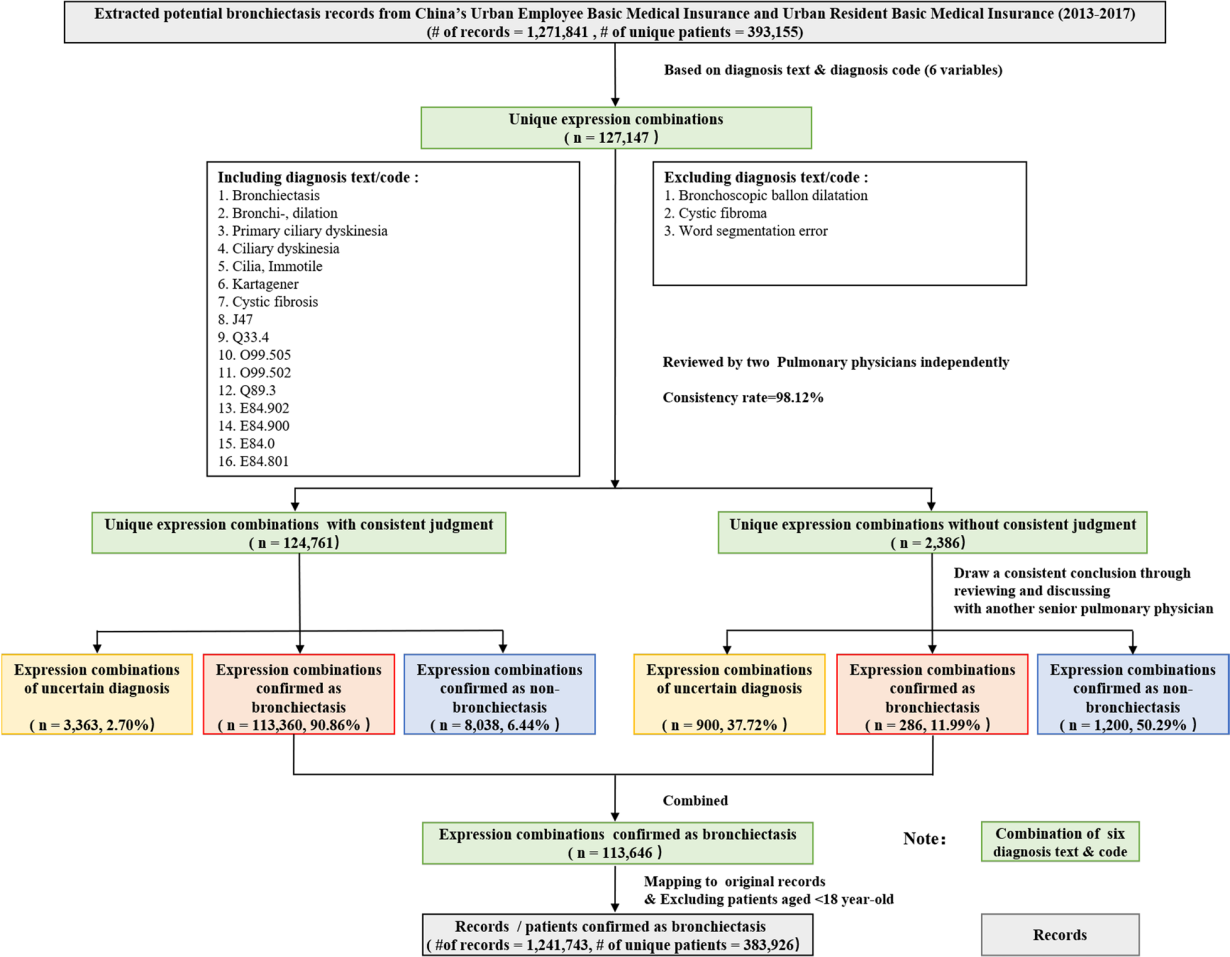
Flow chart of case ascertainment for bronchiectasis

We identified bronchiectasis using ICD-10 code and medical terms in Chinese. To avoid missing cases when using the medical terms in Chinese, we constructed a fuzzy string matching algorithm to extract potential bronchiectasis cases from the database. Then, two pulmonary physicians performed manual verification of the diagnostic text. The flow-chart for the case ascertainment was shown in Fig. 1. The inclusion criteria for targeted cases were defined as patients with (1) bronchiectasis; (2) bronchi-, dilation; (3) primary ciliary dyskinesia; (4) ciliary dyskinesia; (5) cilia, Immotile; (6) Kartagener; (7) cystic fibrosis; or (8) ICD-10 (J74/Q33.4/O99.505/O99.502/Q89.3/E84.902/E84.900/E84.0/E84.801). The exclusion criteria were cases with (1) bronchoscopic ballon dilatation; (2) cystic fibroma; or (3) word segmentation error (such as bronchitis, dilation). Inconsistent judgements were reviewed and discussed with another senior pulmonary physician. Finally, 383,926 patients aged ≥ 18 years were identified and included as cases of bronchiectasis in our study.

### Statistical analysis

National prevalence for each of the 5 years from 2013 to 2017 was calculated. Prevalence was stratified by gender and age (18–29, 30–39, 40–49, 50–59, 60–69, 70–79, and ≥ 80 years). Prevalence was calculated using a two-stage approach. Details of the method to calculate the prevalence was provided in the Additional file 1. We firstly calculated age- and sex- specific prevalence in each province. The denominator used to calculate the annual prevalence of bronchiectasis was the total number of populations in UEBMI and URBMI in each province during the year. The numerator was the number of patients with bronchiectasis estimated from the denominator population in each province, taking into account missing data. The total enrolled population in each province could be divided into three groups: subjects with no records of a medical claim (*N*_*1*_), subjects with complete information on diagnosis in claim records (*N*_*2*_), and subjects with claim records but with missing diagnostic information (*N*_*3*_). The observed patients (*M*_*1*_) with bronchiectasis were from *N*_*2*_, whereas the number of cases (*M*_*2*_) in *N*_*3*_ was estimated using a method based on Poisson regression with 10 times of multiple imputations.

In the second stage, a random-effects meta-analysis was used to pool the province-specific estimates to calculate the national average estimates. We checked that the variance of province-specific estimates was stable using the Freeman-Tukey double arcsine transformation [15].

Prevalence was expressed as per 100,000 person-year at risk, and 95% confidence intervals (CIs) were also calculated assuming a Poisson distribution. Age-adjusted rates at national level were calculated using China 2010 census data. Sensitivity analyses were conducted to assess the robustness of the results: (1) including only observed cases to assess the lower bounds of the rates and (2) excluding the top 10% of provinces ranked by the missingness of diagnostic information.

We also calculated bronchiectasis associated disease burden in each year, including total costs per patient, and inpatient costs per patient, average number of visits, hospitalizations and length of stay. The costs in our study were categorized as diagnosis related cost (including computerized tomography, CT and laboratory examination), surgical treatment and pharmaceutical treatment. The per-capita total cost for patients with bronchiectasis was the sum of total cost of all diseases after diagnosis of bronchiectasis in all patients divided by the total number of patients, while the per-capita inpatient costs for patients with bronchiectasis was the sum of total cost of all inpatients divided by the number of inpatients. Cost for bronchiectasis per se was considered to be the total cost when the disease name or ICD code of bronchiectasis was included in the diagnosis text. Costs were discounted by consumer price index (CPI) in each year to 2017 and converted into US dollars based on the 2017 RMB to US dollar exchange rate (period average). The CPI and exchange rate were from 2018 China statistical yearbook. The average number of visits or hospitalizations was the result of total number of visits or hospitalizations to the total number of patients or inpatients. And the average length of hospital stay was the sum of the length of stay of all inpatients divided by the total number of inpatients.

All statistical analyses used Stata version 15.0.

## Results

Of the 380.00 million residents aged 18 years and older in 23 provinces in UEBMI and URBMI databases (Additional file 1: Table S1), a total of 383,926 patients with bronchiectasis were identified. Females accounted for 51.57% of the patients. The mean age at the diagnosis was 59.52 ± 15.00 years. Bronchiectasis was most common in persons aged 60–69 years (26.59%), followed by those aged 50 to 59 years (21.62%). (Table 1).

**Table 1:** Selected characteristics for bronchiectasis patients grouped by sex

		Total	Male	Female
Number		383,926	185,751	197,973
Age, y	Mean (SD)	59.52 (15.00)	60.48 (15.11)	58.62 (14.83)
Age group, n (%)	18–29	12,857 (3.35)	5,935 (3.20)	6,917 (3.49)
	30–39	29,714 (7.74)	13,275 (7.15)	16,431 (8.30)
	40–49	52,849 (13.77)	24,176 (13.02)	28,653 (14.47)
	50–59	82,994 (21.62)	37,362 (20.11)	45,578 (23.02)
	60–69	102,082 (26.59)	49,527 (26.66)	52,480 (26.51)
	70–79	69,490 (18.10)	36,773 (19.80)	32,686 (16.51)
	≥ 80	33,940 (8.83)	18,703 (10.06)	15,228 (7.70)

*SD* standard deviation. 202 patients (0.05%) had missing data for gender

### Bronchiectasis prevalence

**Fig. 2:**
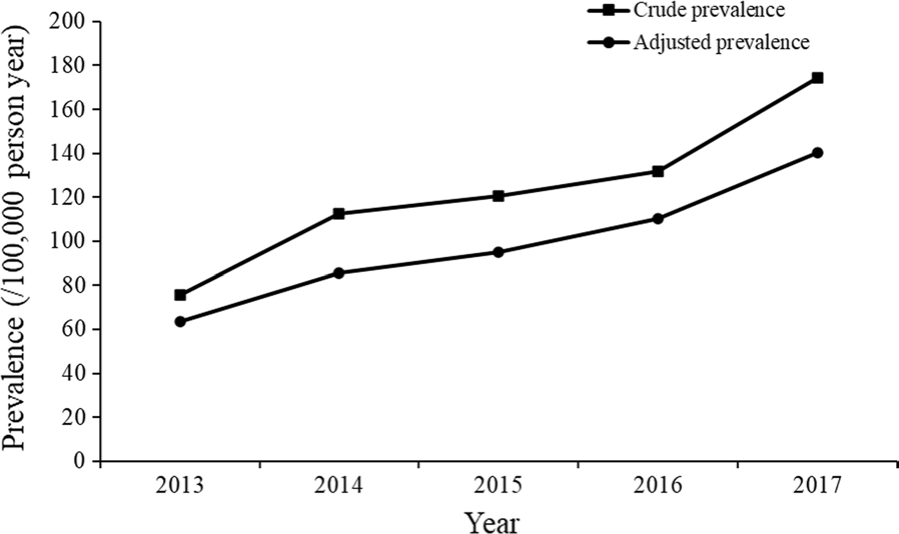
Crude prevalence, adjusted prevalence of bronchiectasis. (units: /100,000 person-years)

Between 2013 and 2017, the total prevalence of bronchiectasis in those aged 18 years and over increased from 75.48 per 100,000 (95% CI 62.26–88.69) to 174.45 per 100,000 (95% CI 137.02–211.88). Similar trends were observed for both genders. For males, the prevalence was 73.42 per 100,000 (95% CI 60.57–86.28) in 2013 and increased to 175.03 per 100,000 (95% CI 137.62–212.43) in 2017, and for females, the prevalence was 76.12 per 100,000 (95% CI 61.51–90.73) in 2013 and rose to 173.60 per 100,000 (95% CI 132.46–214.74) in 2017 (Table 2). Standardized to the China 2010 census population, the total age-adjusted prevalence for bronchiectasis varied from 63.40 (95% CI 47.88–81.12) per 100,000 in 2013 to 140.45 (95% CI 100.82–186.68) per 100,000 in 2017 (Fig. 2).

**Table 2:** Crude prevalence of bronchiectasis grouped by sex and age group (units: /100,000 person-year)

		2013	2014	2015	2016	2017
Total		75.48 (62.26,88.69)	112.61 (89.15,136.08)	120.41 (92.73,148.09)	131.97 (103.27,160.66)	174.45 (137.02,211.88)
Sex	Male	73.42 (60.57,86.28)	109.71 (86.89,132.53)	122.03 (95.97,148.09)	131.20 (104.08,158.32)	175.03 (137.62,212.43)
	Female	76.12 (61.51,90.73)	115.45 (90.55,140.34)	119.19 (89.87,148.51)	133.13 (102.45,163.80)	173.60 (132.46,214.74)
Age group	18–29	14.66 (10.90,18.97)	24.30 (16.23,33.98)	21.74 (14.47,30.48)	22.82 (15.60,31.42)	26.80 (17.52,38.03)
	30–39	37.31 (28.83,46.89)	56.31 (40.91,74.15)	56.74 (40.72,75.42)	61.23 (45.20,79.69)	76.10 (53.03,103.31)
	40–49	52.36 (39.60,66.88)	73.20 (53.09,96.53)	77.07 (53.76,104.57)	84.35 (61.53,110.75)	109.86 (78.53,146.44)
	50–59	89.41 (67.11,114.88)	114.44 (83.58,150.13)	127.21 (88.99,172.22)	143.66 (105.78,187.32)	183.22 (132.56,242.05)
	60–69	134.40 (102.07,171.18)	170.72 (125.14,223.35)	199.86 (141.38,268.42)	240.79 (180.53,309.69)	308.09 (228.15,399.97)
	70–79	217.52 (164.32,278.15)	285.42 (211.02,371.00)	344.38 (245.41,460.00)	389.35 (288.75,504.89)	500.46 (367.77,653.42)
	≥ 80	242.09 (179.48,314.03)	353.79 (236.41,494.66)	436.61 (294.08,607.07)	453.54 (311.16,622.52)	588.80 (402.49,810.20)

**Fig. 3:**
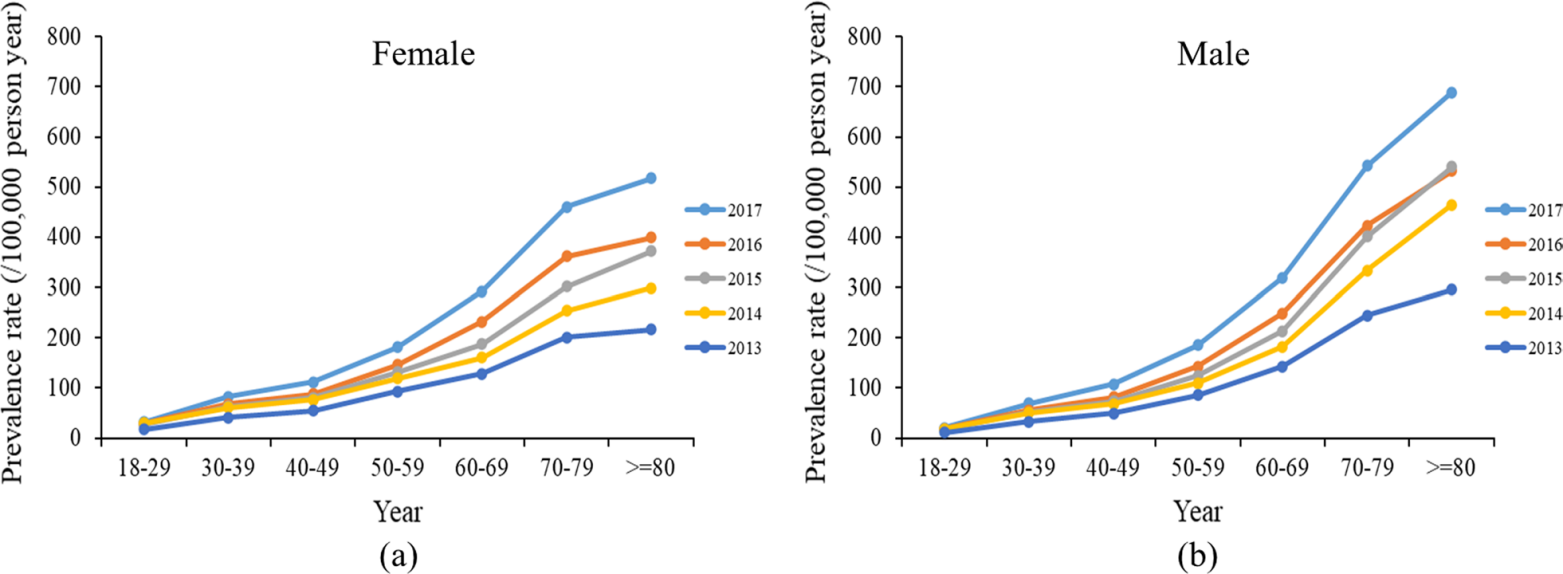
Crude prevalence of bronchiectasis in population grouped by sex–age group (units: /100,000 person-years). **a** Trends in bronchiectasis prevalence for female. **b** Trends in bronchiectasis prevalence for male

Prevalence increased with age, with the highest prevalence in those aged over 80 years and the lowest in those aged 18–29 years (Table 2). Furthermore, the increase was more pronounced for patients aged over 50 years in both genders from 2013 to 2017 (Additional file 1: Table S2 and Fig. 3).

### Disease burden of bronchiectasis

The per-capita total cost in each year for patients with bronchiectasis increased about 2.18-fold from 3537.83 US dollars in 2013 to 7696.54 US dollars in 2017, and the per-capita inpatient cost in each year increased 1.83-fold from 5959.50 US dollars in 2013 to 10,917.07 US dollars in 2017. However, the per-capita total cost in each year for bronchiectasis per se and the per-capita inpatient cost in each year for bronchiectasis per se fluctuated narrowly, from $1650.26 to $2352.34, and from $3023.05 to $3470.86, respectively. This resulted in a decrease of their proportions in the overall cost during the 5 years, from 46.65 to 30.56% and from 50.73 to 31.79%, respectively (Table 3), indicating that the significantly increased costs for the patients were mostly driven by underlying diseases and comorbidities.

**Table 3:** Cost and disease burden of bronchiectasis in urban China in 2013 ~ 2017 (units: US dollars)

	2013	2014	2015	2016	2017
Total cost (outpatients and inpatients)					
Per-capita total cost for patients with bronchiectasis	3537.83	4938.52	5040.17	5654.46	7696.54
Per-capita total cost for bronchiectasis per se	1650.26	2243.34	1697.81	1712.52	2352.34
Proportion of total costs (%)	46.65	45.43	33.69	30.29	30.56
Inpatient cost (only for inpatients)					
Per-capita inpatient costs for patients with bronchiectasis	5959.50	8667.70	8082.50	8120.72	10,917.07
Per-capita inpatient cost for bronchiectasis per se	3023.05	4568.82	3106.71	2829.15	3470.86
Proportion of inpatient costs (%)	50.73	52.71	38.44	34.84	31.79
Average number of visits for bronchiectasis	1.96	2.13	1.97	1.98	2.11
Average number of hospitalizations for bronchiectasis	1.21	1.24	1.20	1.21	1.21
Average length of hospital stay for bronchiectasis (day)	12.03	11.21	10.71	10.28	10.17

During the study period, the average number of visits for bronchiectasis was stable over 2013 ~ 2017 (from 1.96 to 2.13 times), and so the average number of hospitalizations (1.20 to 1.24 times). The average length of hospital stays varied from 10.17 to 12.03 days (Table 3).

### Sensitivity analysis

By considering only observed cases, the lower bound of the national prevalence rose from 32.39 per 100,000 (95% CI 24.54–40.24) to 109.89 per 100,000 (95% CI 82.44–137.35). The results calculated by excluding the top 10% of provinces (Shandong and Xinjiang) with missing diagnostic information ranged from 66.44 per 100,000 (95% CI 52.86–80.02) to 179.90 per 100,000 (95% CI 133.37–226.43), which showed the similar trend as demonstrated by the main results above (Additional file 1: Table S3).

## Discussion

Using nationally representative databases covering approximately 0.5 billion urban residents in 2013–2017, this study found that the prevalence of bronchiectasis in urban adults (aged ≥ 18 years) in China increased 2.31-fold during the study period. The prevalence of bronchiectasis was significantly higher and increased more markedly for populations over 50 years old. At the same time, the per-capita total cost and hospitalization cost of patients with bronchiectasis increased 2.18- and 1.83-fold respectively, while those for bronchiectasis per se fluctuated in a narrow range, indicating that the significantly increased economic burden was mostly driven by underlying diseases and comorbidities.

The overall prevalence of bronchiectasis in urban adults (aged ≥ 18 years) in China increased with an average annual rate of 32.8% from 2013 (75.48 per 100,000 person-years) to 2017 (174.45 per 100,000 person-years). Compared to data from high-income countries [2–4, 6], the present study indicated a similarly increasing trend, but by a greater magnitude. Before our study period, the average annual rate of increase in prevalence of bronchiectasis in the UK was 6.8% from 2004 (350.5 per 100,000 person-year in women, 301.2 per 100,000 person-year in men) to 2013 (566.1 per 100,000 person-year in women, 485.5 per 100,000 person-year in men) [2], and in Germany the rate was about 6% from 2009 (52.5 per 100,000 person-year) to 2013 (64.9 per 100,000 person-year). During the same study period, the average annual rate of increase in prevalence of bronchiectasis in Germany was about 12% from 2013 (64.9 per 100,000 person-year) to 2017 (94.8 per 100,000 person-year) [3] and in South Korea the rate was 2.2% from 2013 (441 per 100,000 person-years) to 2017 (480 per 100,000 person-years).[4] The reasons for increasing prevalence of bronchiectasis worldwide are largely unknown. However, we agree that improved diagnosis with widespread use of CT scans to assess patients with respiratory symptoms is one of the most likely reasons [1, 2, 10]. For example, the annual percentage change (APC) of bronchiectasis was 8.74%, representing a significant increase in bronchiectasis from 2000 through 2007, while the APC for thoracic CT scans increased by 10.3% per year during the same period in the US [10]. Although the availability of CT scanners in China was far behind the average level of availability in Organization for Economic Co-operation and Development (OECD) countries, the annual growth rate of CT scanners was much higher than that in OECD countries between 2005 and 2013 [16]. Data on CT application in hospitals from a minority region in southern China also showed an average annual increase of about 6% from 2013 to 2015 [17]. In addition, the implementation of Chinese bronchiectasis guidelines (Expert consensus on diagnosis and treatment of adult bronchiectasis 2012) and policies promoting the diagnosis and treatment of chronic airway diseases nationwide could also contribute to improved diagnosis and management of bronchiectasis [18]. Finally, the dramatically increasing prevalence of COPD and asthma may account for more cases of associated bronchiectasis. For example, the prevalence of COPD in Chinese populations aged ≥ 40 years increased from 8.2% to 13.7% in 10 years [19, 20]. Based on the above considerations, we speculate that the prevalence and medical burden of bronchiectasis will continue to increase in China.

The finding that the prevalence of bronchiectasis in men and women increased with increasing age in our study was consistent with data from other countries [2, 4–6, 10] except that the prevalence rate showed a slight decline in the > 80 years group in some studies [2, 5, 10]. In addition, the increase was more pronounced for patients aged over 50 years in both genders. Previous studies showed that chronic productive cough was present in 98% of patients with bronchiectasis, over 59–80% of whom had chronic respiratory symptoms from childhood [21, 22] or youth [23], but most of these patients were diagnosed with bronchiectasis 12 to more than 30 years after symptom onset [22–24]. There was a marked delay from symptom onset to diagnosis of bronchiectasis. Bronchiectasis patients with symptom onset in childhood described their symptoms becoming worse after the age of 50 years [22]. The aggravation of symptoms may promote the patient to seek medical care and hence the diagnosis of bronchiectasis. In addition, there was a bi-modal distribution of the onset age of productive cough; most common in the first 15 years of life followed by over the age of 50 years [21].

With the increase of prevalence, bronchiectasis brings huge medical and economic burden to the society. In our study, the mean annual visit for bronchiectasis was around 2.0 times, while the yearly hospitalization was 1.2 times, which were comparable to data (0.3–1.3) from a systematic literature review [25]. The per-capita total cost and hospitalization cost for our patients with bronchiectasis increased 2.18- and 1.83-fold respectively during the study period, while the per-capita total cost and hospitalization cost for bronchiectasis per se only fluctuated in a narrow range. The finding that the overall medical cost was increasing while the cost for bronchiectasis was relatively stable indicated that the costs for underlying diseases or comorbidities of patients with bronchiectasis increased significantly during the study period. In recent years, the higher incidence of comorbidities such as cardiovascular diseases in patients with bronchiectasis have received much attention. Studies demonstrated that the prevalence of coronary heart disease (CHD) and stroke was higher in people with bronchiectasis compared with those without bronchiectasis, after adjusting for confounding factors [26], and these comorbidities were associated with bronchiectasis severity [27]. Furthermore, bronchiectasis is associated with a variety of underlying diseases. Studies in China showed that there were more than 10 underlying causes for bronchiectasis, including infectious diseases, autoimmune diseases, asthma, COPD, and allergic bronchopulmonary aspergillosis [28, 29]. Since new treatments were emerging [30, 31] and the annual cost of these therapies was increasing [32], the economic burden of some underlying diseases of bronchiectasis was increasing during the study period. In addition, studies in recent years also showed that the presence of bronchiectasis was associated with longer length of hospital stay and higher costs in patients with asthma or COPD [33–35]. These results indicate that clinicians and health policy-makers should pay more attention to this increasingly important respiratory disease with multiple comorbidities, and to explore effective ways to improve diagnosis and management of the underlying diseases or comorbidities for reducing morbidity and mortality.

Taken together, the rising prevalence of bronchiectasis, along with the increase in bronchiectasis-associated costs, is becoming a substantial challenge and economic burden for the whole society in China. Besides, from this study, we predict that the prevalence of bronchiectasis will increase dramatically from about 231.64 per 100,000 in 2020, to 340.29 per 100,000 in 2025, and 448.93 per 100,000 in 2030. Our findings highlight the urgent need for prevention and optimal management of this common airway disease in the world’s largest population.

### Strengths and limitations

Our study used a large, nationally representative sample of the Chinese mainland urban population, to provide a reliable estimate of the prevalence and costs of bronchiectasis. The large number of urban adult patients in the cohort across the country, the diagnosis of bronchiectasis in medical records and the long study period enabled us to make accurate estimates of bronchiectasis prevalence stratified by age groups.

This study also has several limitations. First, the database did not cover rural areas, which have a different insurance system. However, the combined basic population structure was close to the distribution in the China 2010 population census data. Second, missing diagnostic variables could have affected the estimates. However, sensitivity analyses were used to explore the potential influence of these on the estimations [36].Third, case ascertainment was limited, because the basic medical insurance databases did not provide information on laboratory data, imaging results and etiology data. We did not have access to CT scan data and were not sure whether in each case of the diagnosis of bronchiectasis was made according to current guidelines and whether the underlying etiologies were sought. The data used in this study were summarized data due to privacy protection, and thus we were also unable to access the individual data and contact patients directly to obtain additional information. Fourth, we only identified cases with bronchiectasis as the primary diagnosis and as the first two secondary diagnoses in the medical records, which may lead to an underestimation of the prevalence of the disease.

## Conclusion

This research is the first nationwide, population-based study to investigate the prevalence of bronchiectasis in urban adults in mainland China. The prevalence of bronchiectasis increased 2.31-fold from 2013 to 2017. Although the medical cost for bronchiectasis per se fluctuated narrowly, the cost for comorbidities or underlying diseases increased markedly during the study period. The results suggest that optimal managements for preventing exacerbations and comorbidities are needed to reduce the morbidity and mortality of this common airway disease.

## Supplementary Information

**Additional file 1:** Strategy used in the estimation of numerator. **Table S1.** Basic characteristics of population in 23 provinces of China during 2013–2017. **Table S2.** Crude prevalence of bronchiectasis in population grouped by sex–age group (unit:/100,000 person-years). **Table S3.** Results of sensitivity analysis for prevalence (unit:/100,000 person-years).

## Data Availability

Summarized health data on bronchiectasis can be accessed by contacting the National Insurance Claims for Epidemiological Research (NICER) Group, School of Public Health, Peking University. Contact email: 0016163159@bjmu.edu.cn.

## Abbreviations

UEBMI: Urban employee basic medical insurance
URBMI: Urban resident basic medical insurance
ICD: International Classification of Diseases
CI: Confidence interval
SD: Standard deviation
CPI: Consumer price index
APC: Annual percentage change
COPD: Chronic obstructive pulmonary disease
OECD: Organization for Economic Co-operation and Development
CHD: Coronary heart disease
CT: Computerized tomography
