# Supplementary material for: Increasing prevalence and burden of bronchiectasis in urban Chinese adults, 2013–2017: a nationwide population-based cohort study
Source: Respir Res. 2022 May 4;23:111. doi: 10.1186/s12931-022-02023-8 (PMC9066779; doi:10.1186/s12931-022-02023-8)
Supplement: Supplementary file 1 — Additional file 1: Strategy used in the estimation of numerator. Table S1. Basic characteristics of population in 23 provinces of China during 2013–2017. Table S2. Crude prevalence of bronchiectasis in population grouped by sex–age group (unit:/100,000 person-years). Table S3. Results of sensitivity analysis for prevalence (unit:/100,000 person-years). [file 12931_2022_2023_MOESM1_ESM.pdf]

Supplementary (online) tables and figures

Additional file 1
Strategy used in the estimation of numerator

The denominator used to calculate the annual prevalence of bronchiectasis is defined as the total number of subjects in UEBMI and URBMI in each provincial age and sex-specific group during the year. In the pilot analysis for the national insurance database, we found that the diagnostic information may be missing, often due to administrative reasons. The total enrolled population in each subgroup (N_{ij}) can be classified into three categories: subjects with no records of any medical claim (N_{1ij}), subjects with complete information on diagnosis in claim records (N_{2ij}), and subjects with claim records but missing diagnostic information (N_{3ij}). Complete-case analysis was not feasible in this case due to the existence of the population without any claim (i.e. healthy population which accounts for a significant proportion of the population). Ignoring the missingness will lead to the underestimation of the prevalence. Therefore, we design the following imputation algorithm to take account for the influence caused by missing information on the diagnosis.

The total population can be divided as shown in the following table.

	Case	Non-case	Total
Subjects with diagnostic information	a_{ij}	b_{ij}	N_{2ij}
Subjects without diagnostic information	c_{ij}	d_{ij}	N_{3ij}
Subjects without any claim	0	e_{ij}	N_{1ij}
**Population in each subgroup**	$\mathbf{M_{ij}}$	$\mathbf{N_{ij} - M_{ij}}$	$\mathbf{N_{ij}}$

where i denoted the age- and sex- subgroup and j represented the province. Patients with bronchiectasis, noted as M_{ij}, is referred to the nominator to calculate the prevalence. Prevalence can then be calculated as

$$\mathbf{I_{ij}} = \frac{\mathbf{a_{ij}} + \mathbf{c_{ij}}}{\mathbf{a_{ij}} + \mathbf{b_{ij}} + \mathbf{c_{ij}} + \mathbf{d_{ij}} + \mathbf{e_{ij}}} \quad \text{(A1)}$$

Considering the fact that the missingness of diagnosis was often due to administrative reasons which should not be specific to the disease of bronchiectasis, we assumed the proportion of bronchiectasis cases in subjects with or without diagnostic information were equal, i.e.

$$\frac{\mathbf{a_{ij}}}{\mathbf{a_{ij}} + \mathbf{b_{ij}}} = \frac{\mathbf{c_{ij}}}{\mathbf{c_{ij}} + \mathbf{d_{ij}}} \quad \text{(A2)}$$

The number of cases in subjects without diagnostic information (c_{ij}) was then expressed as

$$\mathbf{c_{ij}} = \frac{\mathbf{a}}{N_{2ij}} \times N_{3ij} = r_{ij} N_{3ij} \quad \text{(A3)}$$

where r_{ij} was referred to the prevalence rate in subjects with complete information on diagnosis. In each province, we fit the Poisson regression model as

$$r_j = e^{\beta_0 + \beta_1 \text{Age} + \beta_1 \text{Sex} + \beta_2 \text{Year} + \beta_2 \text{Type}} \quad \text{(A4)}$$

where Type was referred to the type of the insurance (i.e. UEBMI or URBMI). The expected number of cases in each subgroup (including both subjects with and without diagnostic information) can then be calculated based on the predictions from the Poisson model. Moreover, to account for the uncertainty from the model prediction, we generated 10 estimated number of cases sampled from the distribution. For the crude analysis, the province-specific prevalence was directly calculated using the equation A1 where each element was the summation of all age- and sex- subgroups, e.g. $a_j = \sum_i a_{ij}$. Finally, the prevalence for each province or subgroup was calculated by pooling ten estimates using Rubin's Rule.

Table S1. Basic characteristics of population in 23 provinces of China during 2013–2017（units: million）.

		Total	UEBMI	URBMI
Total Number, n (%)		380·00	175·75	204·25
Age, y	Mean (SD)	44·08 (17·21)	43·80 (15·37)	44·32 (18·65)
Age group, n (%)	18-29	97·11 (25·56)	36·12 (20·55)	60·99 (29·86)
	30-39	73·48 (19·34)	44·51 (25·32)	28·97 (14·18)
	40-49	73·50 (19·34)	38·40 (21·85)	35·10 (17·18)
	50-59	58·17 (15·31)	26·81 (15·25)	31·36 (15·35)
	60-69	43·45 (11·44)	17·36 (9·88)	26·09 (12·78)
	70-79	21·79 (5·74)	8·33 (4·74)	13·46 (6·59)
	≥80	12·50 (3·27)	4·22 (2·41)	8·28 (4·06)
Gender, n (%)	Male	184·78 (48·63)	78·76 (44·82)	106·02 (51·91)
	Female	195·22 (51·37)	96·99 (55·18)	98·23 (48·09)

Abbreviations: UEBMI, Urban Employee Basic Medical Insurance; URBMI, Urban Residence Basic Medical Insurance. SD, standard deviation.

Table S2. Crude prevalence of bronchiectasis in population grouped by sex–age group (unit:/100,000 person-years).

		2013	2014	2015	2016	2017
Female	18-29	17·22 (12·50,22·70)	29·69 (18·52,43·48)	26·71 (16·97,38·67)	28·18 (18·66,39·67)	31·77 (20·38,45·67)
	30-39	40·79 (30·97,51·95)	61·11 (44·54,80·30)	62·45 (44·03,84·08)	67·77 (48·52,90·23)	83·09 (57·33,113·61)
	40-49	54·32 (40·67,69·95)	76·28 (54·67,101·47)	80·87 (55·65,110·77)	87·43 (61·93,117·30)	112·01 (78·29,151·74)
	50-59	92·76 (68·08,121·25)	118·58 (83·80,159·36)	131·09 (89·00,181·29)	145·52 (105·63,191·77)	180·90 (128·20,242·65)
	60-69	128·30 (92·99,169·28)	160·34 (110·85,218·92)	187·96 (128·22,259·06)	231·83 (170·74,302·21)	292·73 (210·38,388·60)
	70-79	200·91 (145·57,265·12)	253·68 (177·94,342·77)	301·88 (203·85,419·02)	361·92 (257·37,484·16)	460·63 (322·78,622·79)
	≥80	216·94 (153·34,291·52)	299·09 (190·56,431·90)	372·05 (234·70,540·76)	399·46 (253·62,578·12)	517·88 (326·18,753·42)
Male	18-29	11·42 (8·65,14·56)	18·32 (12·36,25·46)	16·82 (11·27,23·48)	17·76 (12·05,24·58)	20·40 (13·17,29·20)
	30-39	33·14 (25·80,41·39)	50·16 (37·71,64·38)	51·02 (37·38,66·77)	56·08 (41·09,73·40)	69·52 (48·21,94·72)
	40-49	48·93 (36·98,62·55)	68·47 (49·96,89·89)	73·22 (52·52,97·34)	81·54 (60·41,105·81)	107·04 (76·74,142·36)
	50-59	85·44 (65·17,108·44)	110·06 (80·45,144·30)	123·74 (88·48,164·90)	142·70 (107·38,183·02)	185·22 (134·16,244·48)
	60-69	142·52 (109·22,180·25)	181·95 (135·53,235·17)	213·20 (152·92,283·45)	249·06 (188·21,318·40)	319·72 (234·32,418·32)
	70-79	244·28 (186·63,274·46)	334·43 (251·07,429·69)	401·37 (297·47,520·73)	423·50 (324·70,535·34)	542·63 (398·78,708·44)
	≥80	295·83 (215·02,389·46)	464·76 (319·68,636·76)	540·25 (374·48,736·13)	532·01 (380·11,709·22)	689·20 (476·77,940·34)

Table S3. Results of sensitivity analysis for prevalence (unit:/100,000 person-years).

	2013	2014	2015	2016	2017
Using only observed cases	32·39 (24·54,40·24)	49·73 (36·77,62·68)	59·12 (41·29,76·95)	79·33 (57·11,101·54)	109·89 (82·44,137·35)
Excluding the top 10% of provinces with missing diagnostic information [a]	66·44 (52·86,80·02)	105·69 (78·8,132·58)	123·45 (89·95,156·94)	135·52 (101·14,169·90)	179·90 (133·37,226·43)

a: Shandong and Xinjiang provinces were excluded; the above table cells were the prevalence and their 95% confidence intervals.
